# Microarray analysis of a salamander hopeful monster reveals transcriptional signatures of paedomorphic brain development

**DOI:** 10.1186/1471-2148-10-199

**Published:** 2010-06-28

**Authors:** Robert B Page, Meredith A Boley, Jeramiah J Smith, Srikrishna Putta, Stephen R Voss

**Affiliations:** 1 Department of Biology and Spinal Cord and Brain Injury Research Center, University of Kentucky, Lexington, KY 40506, USA; 2 Benaroya Research Institute, Seattle, WA 98101, USA

## Abstract

**Background:**

The Mexican axolotl (*Ambystoma mexicanum*) is considered a hopeful monster because it exhibits an adaptive and derived mode of development - paedomorphosis - that has evolved rapidly and independently among tiger salamanders. Unlike related tiger salamanders that undergo metamorphosis, axolotls retain larval morphological traits into adulthood and thus present an adult body plan that differs dramatically from the ancestral (metamorphic) form. The basis of paedomorphic development was investigated by comparing temporal patterns of gene transcription between axolotl and tiger salamander larvae (*Ambystoma tigrinum tigrinum*) that typically undergo a metamorphosis.

**Results:**

Transcript abundances from whole brain and pituitary were estimated via microarray analysis on four different days post hatching (42, 56, 70, 84 dph) and regression modeling was used to independently identify genes that were differentially expressed as a function of time in both species. Collectively, more differentially expressed genes (DEGs) were identified as unique to the axolotl (*n *= 76) and tiger salamander (*n *= 292) than were identified as shared (*n *= 108). All but two of the shared DEGs exhibited the same temporal pattern of expression and the unique genes tended to show greater changes later in the larval period when tiger salamander larvae were undergoing anatomical metamorphosis. A second, complementary analysis that directly compared the expression of 1320 genes between the species identified 409 genes that differed as a function of species or the interaction between time and species. Of these 409 DEGs, 84% exhibited higher abundances in tiger salamander larvae at all sampling times.

**Conclusions:**

Many of the unique tiger salamander transcriptional responses are probably associated with metamorphic biological processes. However, the axolotl also showed unique patterns of transcription early in development. In particular, the axolotl showed a genome-wide reduction in mRNA abundance across loci, including genes that regulate hypothalamic-pituitary activities. This suggests that an axolotls failure to undergo anatomical metamorphosis late in the larval period is indirectly associated with a mechanism(s) that acts earlier in development to broadly program transcription. The axolotl hopeful monster provides a model to identify mechanisms of early brain development that proximally and ultimately affect the expression of adult phenotypes.

## Background

Darwin [1] proposed that evolution by natural selection is a gradual process that results in continuous phenotypic variation among species. However, there are many examples where discontinuous phenotypes are observed among related species and thus appear to evolve rapidly. That evolution could suddenly "leap forward" led to extensions of Darwin's theory to account for the rapid origin of novel phenotypes. One very old idea is that novel and dramatically different phenotypes originate via saltational evolution from mutations of genes that regulate key developmental or physiological processes during ontogeny. In particular, Goldschmidt [2] proposed that mutations occasionally yield individuals within populations that deviate radically from the norm and referred to such individuals as "hopeful monsters". If the novel phenotypes of hopeful monsters arise under the right environmental circumstances, they may become fixed, and the population will found a new species. While this idea was discounted during the Modern Synthesis [3], aspects of the hopeful monster hypothesis have been substantiated in recent years. For example, it is clear that dramatic changes in phenotype can occur from few mutations of key developmental genes and phenotypic differences among species often map to relatively few genetic factors [4-8]. These findings are motivating renewed interest in the study of hopeful monsters and the perspectives they can provide about the evolution of development [9,10]. In contrast to mutants that are created in the lab, hopeful monsters have been shaped by natural selection and are therefore more likely to reveal mechanisms of adaptive evolution.

At least three lines of evidence led Goldschmidt [2] to cite the Mexican axolotl (*Ambystoma mexicanum*) as one of the original hopeful monsters. First, the axolotl follows a different ontogeny from other closely related tiger salamanders. Whereas some tiger salamanders undergo an obligatory metamorphosis during ontogeny that allows for a transition from an aquatic habitat to a more terrestrial habitat, the axolotl has a non-metamorphic life cycle that is often referred to as paedomorphic [11]. This extreme example of discontinuous phenotypic variation supports a model of evolution by heterochrony: larval morphological traits of ancestral metamorphic forms are observed in the adult stages of derived paedomorphic forms. In the minds of early evolutionary biologists, these patterns were so clearly supportive of heterochrony that the Mexican axolotl became the exemplar of evolution by neoteny [12,13]. The second reason Goldschmidt cited the axolotl was physiological - Huxley [14] had shown that a single molecule - thyroid hormone (TH) - was capable of rescuing metamorphosis in the axolotl. Thus, the axolotl seemed to be an example of evolution waiting around for the right macromutation to happen - simply block a single physiological step in TH regulation and a novel form is originated. The third reason Goldschmidt cited the axolotl was ecological. Previous researchers had noted that the axolotl was endemic to the high quality, permanent aquatic habitats of Xochimilco, which is near present day Mexico City [15]. The evolution of paedomorphosis seemingly allowed the axolotl to exploit an empty niche in an environment that was devoid of predators.

Since Goldschmidt, the axolotl has remained a quintessential hopeful monster [16]. Speculation that the paedomorphic condition of the axolotl could have a simple mechanistic basis was supported when a quantitative trait locus (QTL) was identified for the segregation of metamorphic and paedomorphic phenotypes in interspecific crosses [8,17-20]. Previous physiological studies had also established that axolotls do not produce a sufficient titer of thyroid hormone during larval development to initiate anatomical metamorphosis [[21,22] reviewed in [23-25]]. The evolution of axolotl hypothyroidism is thought to be associated with a mechanism that affects the development and/or function of neuroendocrine axes that regulate the release of thyroid hormone from the thyroid glands [11,26,27]. Conceivably, this mechanism could function during early stages of development or it could function later in the larval period when metamorphosis is initiated. Regardless, whether paedomorphic and metamorphic larvae show similar or different patterns of neurological development and function has not been previously investigated.

In this study, microarray analysis was used to investigate transcription within whole brains (including the pituitary) of the paedomorphic axolotl and a closely related metamorphic species (*A. tigrinum tigrinum*). The primary objective was to identify patterns of gene expression during early ontogeny that could provide new mechanistic insights about paedomorphic and metamorphic modes of development. Transcripts were sampled from both species at four chronologically matched times post hatching to obtain temporal profiles of gene expression during the early larval period and during early stages of morphological metamorphosis in *A. t. tigrinum*. Hundreds of genes showed different or unique patterns of expression between the species, many of which were initiated very early in the larval period and prior to the onset of morphological metamorphosis. The results suggest considerable potential for transcriptional divergence between closely related vertebrate species and highlight the tiger salamander/axolotl model system for examining mechanisms in the developing brain that determine adult phenotypic outcomes.

## Results

### Larval growth and metamorphosis

**Figure 1:**
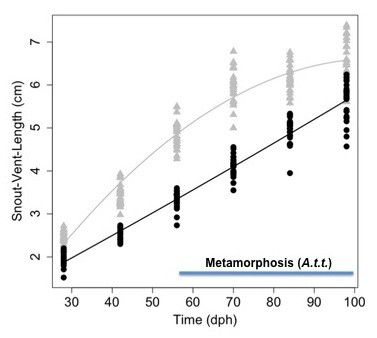
**Growth of larval axolotls and tiger salamanders**. Data points are snout vent length (SVL) measured for individuals at the time of brain tissue collection. Lines were estimated by fitting a general linear model (*R*^*2 *^= 0.957) to SVL data from both species. All terms of the model were significant (p < 0.05; see methods). Axolotl larvae = black circles, tiger salamander larvae = gray triangles. The blue bar shows the time period where anatomical changes consistent with metamorphosis were observed in tiger salamander larvae (*A.t.t*.).

Under normal growth conditions, larvae of metamorphic and paedomorphic species of *Ambystoma *increase in size but only larvae of metamorphic species show changes in morphology (bulging eyes, changes in head shape, reduction of tailfins and gills) that are indicative of anatomical metamorphosis. In this experiment, tiger larvae were larger than axolotls at 28 dph and exhibited higher growth rates early in the larval period (Figure 1). As development proceeded, tiger salamander growth rates decelerated while axolotl growth rates remained constant. None of the tiger larvae showed changes in morphology suggestive of initiation of metamorphosis at the three earliest time points (28, 42, 56 dph). However at 70 dph, 23% (*n *= 30) of tiger larvae showed bulging eyes and subtle changes in head morphology. By 84 dph, all tiger larvae were undergoing anatomical metamorphosis and 20% (*n *= 30) of these had rudiments of gills that were less than 1 mm in length. These larvae, and all larvae that were examined at 98 dph (*n *= 30), were considered metamorphs. Thus, some tiger salamanders initiated anatomical metamorphosis between 56 and 70 dph and most larvae completed metamorphosis between 84 and 98 dph.

### Differentially expressed genes identified independently from axolotls and tiger salamanders

**Figure 2:**
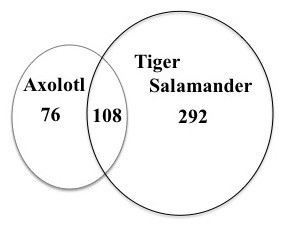
**Venn diagram showing differentially expressed genes (DEGs)**. The figure shows DEGs identified uniquely from axolotls and tiger salamanders, and DEGs in common between the species.

A custom Affymetrix GeneChip [see [28-30]] was used to estimate mRNA abundances at four chronologically matched times (42, 56, 70, and 84 dph) to obtain temporal profiles of gene expression for tiger salamander and axolotl larvae. Statistical and fold-change criteria were then used to identify genes that were differentially expressed within each of the species. Thus, these analyses took the conservative approach of independently identifying genes from each species that were differentially expressed as a function of time and comparing the list of differentially expressed genes (DEGs) from tiger salamander with the list of DEGs identified from axolotl. This approach is conservative because it reduces the risk of identifying false positive expression differences between species that are caused by heterologous hybridization [31]. More than twice as many DEGs were identified from tiger salamander than axolotl larvae (*n *= 400 vs 184) (Figure 2). There was considerable overlap between the two species as more than half (*n *= 108) of the genes identified from axolotls were also identified from tiger salamander larvae. The remaining DEGs changed uniquely in only one species. Thus, many shared and unique DEGs were identified, with more unique DEGs identified from tiger salamander larvae.

**Figure 3:**
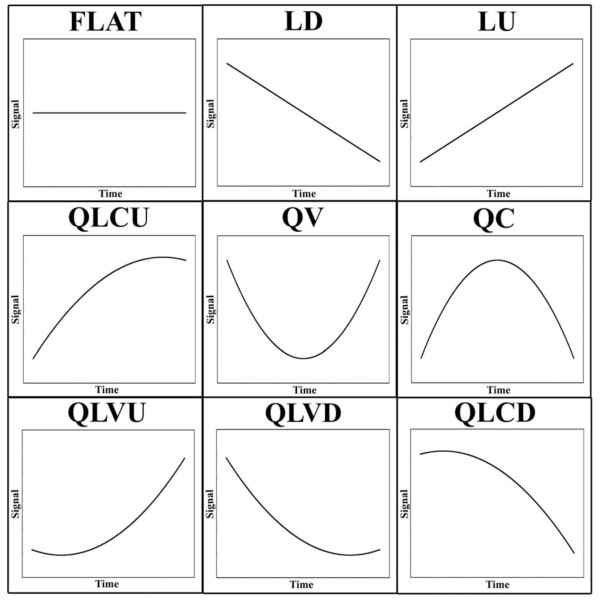
**Regression patterns of differentially expressed genes identified from axolotls and tiger salamanders**. LD = linear down, QLCD = quadratic linear concave down, QLVD = quadratic linear convex down, QV = quadratic convex, LU = linear up, QLVU = quadratic linear convex up, QLCU = quadratic linear concave up, and QC = quadratic concave.

Of the 108 DEGs identified from both species, 60% exhibited the same regression profile and 99% exhibited the same generalized direction of differential expression (*i.e*., up versus down-regulation; Additional File 1). Of the 76 DEGs uniquely identified from axolotls (Additional File 2), 55% showed increasing mRNA abundances (LU, QLVU, or QLCU) during the larval period while 45% showed decreasing abundances (LD, QLVD, or QLCD) (see Figure 3 for acronym definitions). The opposite pattern was observed for the 332 unique tiger salamander DEGs with 55% showing decreasing mRNA abundances, 43% showing increasing abundances, and 1% showing a pattern of transient increase (QC). Only a small percentage (~10%) of shared and unique DEGs exhibited ≥ 1.5 fold difference in mRNA abundance between the earliest time points (42 and 56 dph). However, between the latest time points (70 and 84 dph), ~32% of the shared DEGs, 64% of unique axolotl DEGs, and 61% of unique tiger salamander DEGs showed a ≥ 1.5 fold difference. Thus, abundances of uniquely expressed transcripts tended to increase throughout larval development, showing the largest fold differences at later larval stages.

**Figure 4:**
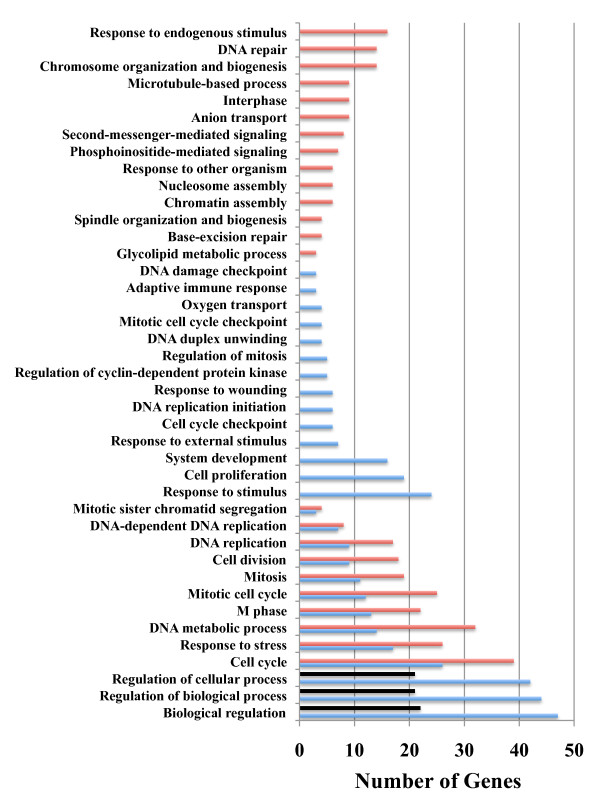
**Gene ontology (GO) terms identified as significantly over-represented**. The figure shows GO terms for differentially expressed genes (DEGs) identified uniquely from axolotls (black bars) and tiger salamanders (red bars), and GO terms for DEGs that were identified in common between the species (blue bars).

The DEGs identified independently from both species that showed significant sequence identity to a human RefSeq protein were assumed to be salamander-human orthologs and were annotated with biological process information from the Gene Ontology (GO) database. Many of the same GO terms were represented among the three DEG lists - the DEGs that were expressed in common between the species (i.e. shared list) and the separate lists of DEGs that were identified uniquely from each species (i.e. axolotl or tiger salamander). The commonly expressed DEGs were statistically associated with 27 terms, and many of these genes are predicted to function in cell cycle processes (Figure 4). Nearly all of the DEGs that annotated to cell cycle terms exhibited decreasing mRNA abundances during larval development in both species, as did six of seven annotated transcription factors. For example, two biomarkers of neural development and differentiation (*sox3, msx1*) were both categorized as LD in axolotl and tiger salamander. Conversely, the majority of DEGs associated with system development were up regulated in both species. These results suggest that some aspects of neural development and function are similarly regulated between axolotl and tiger salamander larvae. This also seems to be true for some but not all hemoglobin loci, and genes that function in stress and immunological pathways.

Relatively few uniquely expressed axolotl genes were identified overall (N = 76) and thus only 3 broad GO terms were identified as statistically enriched (Figure 4): regulation of cellular process (*n *= 21, *p *= 0.004), regulation of biological process (*n *= 21, *p *= 0.011), and biological regulation (*n *= 22, *p *= 0.015). The unique axolotl genes are predicted to function in some but not all of the biological processes observed for the shared DEG list (Additional File 2). For example, six genes that function in apoptosis (*srpbp1*, *anax1, anax5, mtch1*, *gstp1, pim1*) were uniquely identified for axolotls. DEGs known to be associated with vertebrate brain development were identified, including genes that code for extracellular matrix constituents and cell adhesion (*e.g*., *mmp1*, *dcn*, *col1a1, dpt, lgals4*). Also, several biomarkers of mammalian brain pathologies were uniquely up regulated in axolotls (*ctss, ogn, cd69*). These and other uniquely expressed genes may be associated with the axolotl's paedomorphic mode of development.

The larger list of unique DEGs from tiger larvae yielded more biological process annotations and were statistically associated with 23 GO terms (Figure 4)(Additional File 3). As was observed for the shared gene list, the cell cycle GO term was significantly enriched and these genes showed decreasing mRNA abundances during larval development. However, several GO terms were identified that were not represented in the shared or unique axolotl list, including biological processes associated with chromatin organization and biogenesis. For example, *lmx1b *showed a pattern of decreasing transcript abundance, as did several other genes that function in chromatin organization, modification, and gene silencing (e.g. *dnmt1, baz1b, baz1a, smarca5*, *hist1h1b, hist1hbj, hist2h2ac*). It is possible that some of these unique DEGs are associated with the maturation of brain regions that orchestrate metamorphic events. For example, several genes that function to regulate the secretion of hypothalamic, pituitary, and interrenal hormones were uniquely expressed in tiger salamander larvae, including *nr3c2*, *prl*, and *sstr5*. In addition to these genes, *pomc *and *crhr1 *exhibited higher expression levels in tiger salamander larvae (see real-time PCR results below). Thus, the microarray analysis identified expression differences between axolotl and tiger larvae that may correlate with HPI axis regulation and function.

### Direct comparison of transcription between axolotl and tiger salamander

**Figure 5:**
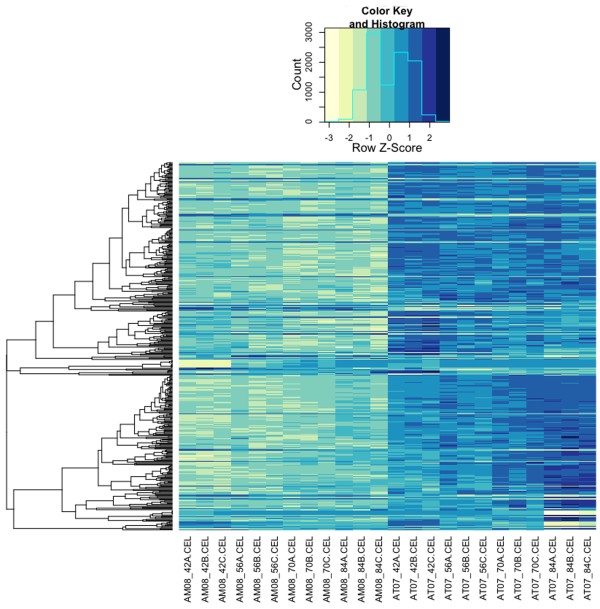
**Heat map of differentially expressed genes**. The figure shows row centered and scaled data from 419 genes identified by directly comparing transcript abundances between axolotls and tiger salamanders. The columns correspond to individual GeneChips with the prefixes AM and AT denoting axolotl GeneChips and tiger salamander GeneChips respectively. Time points correspond to days post hatching and are denoted by numbers followed by a letter (e.g., AM08_42C.CEL corresponds to an RNA pool derived from axolotls sampled at 42 dph). The dendrogram to the left was obtained via hierarchical clustering of a pair-wise distance matrix that was calculated as |1 - *r*|, where *r *= Pearson's correlation coefficient.

To complement the statistical analyses described above, a subset of Affymetrix probesets were identified that could be used to reliably compare transcript abundance estimates directly between the species. Othologous genes from axolotl and tiger salamander were aligned to identify 1320 Affymetrix probe-sets with zero mismatches between the species. Ten of these probesets were identical to other probesets. Of the remaining non-redundant probesets, 31% (*n *= 409) registered statistically distinct expression profiles between the species. This analysis identified some of the unique, temporally regulated DEGs described above, and additionally, genes that exhibited flat regression profiles that differed significantly in elevation between the species. Indeed, 84% (*n *= 343) of the genes identified from this analysis showed higher expression levels throughout the larval period in tiger salamanders (Figure 5; Additional File 4). This list is statistically enriched with genes that function in oxygen/gas transport (*n*= 4, *p *= 0.012), lipid transport (*n *= 6, *p *= 0.035), and heterocycle metabolic processes (*n *= 7, *p *= 0.038). With respect to oxygen transport, several hemoglobin genes (*hbg1*, *hbe1, hbd, hba*) exhibited > 10 fold differences between the species. The complete list of genes that showed higher abundances in tiger salamanders annotate to many different biological processes, including mRNA biosynthesis (*med8, med31, polr2d, taf8, taf12*), protein translation (*eef1g, eif2s1, eif3i*), post-translational modification of proteins (*spop, sumo1, uba2, ube2e3, ebe2i, ube2l3, ube2r2, usp2*), neural function and development (*aldh1a1, bambi, id3, smad5, sigmar1, nr3c2*), stress and immune response (*cat, hmox1, ifi44l, gpx1, oxr1, sftpd*), and hypothalamic-pituitary function (*fst*, *nr3c2, tef*). The relatively few genes (*n *= 41) that showed consistently higher transcript abundances in axolotl larvae also annotate to a number of different biological processes, including DNA metabolism (*kpna2*, *rrm1, rrm2*), cell cycle regulation (*pim3, cdk4, cdc20*), collagen metabolism (*mmp1, mmp9, col3a1, col6a1, col8a1*), neural development (*ndrg2*), and cellular metabolism (*eno1, pgym, fbp2, acy3*). It is unlikely that the interspecific expression differences described above are artifacts of heterologous hybridization because > 80% of the probe-sets were designed from the species (axolotl) that showed consistently lower transcriptional abundances across genes. These results further demonstrate that genes are differentially expressed between the developing brains of axolotl and tiger salamander larvae, and that mRNA abundance is on average higher for tiger salamander orthologs.

### Further investigation of gene expression using qPCR

**Figure 6:**
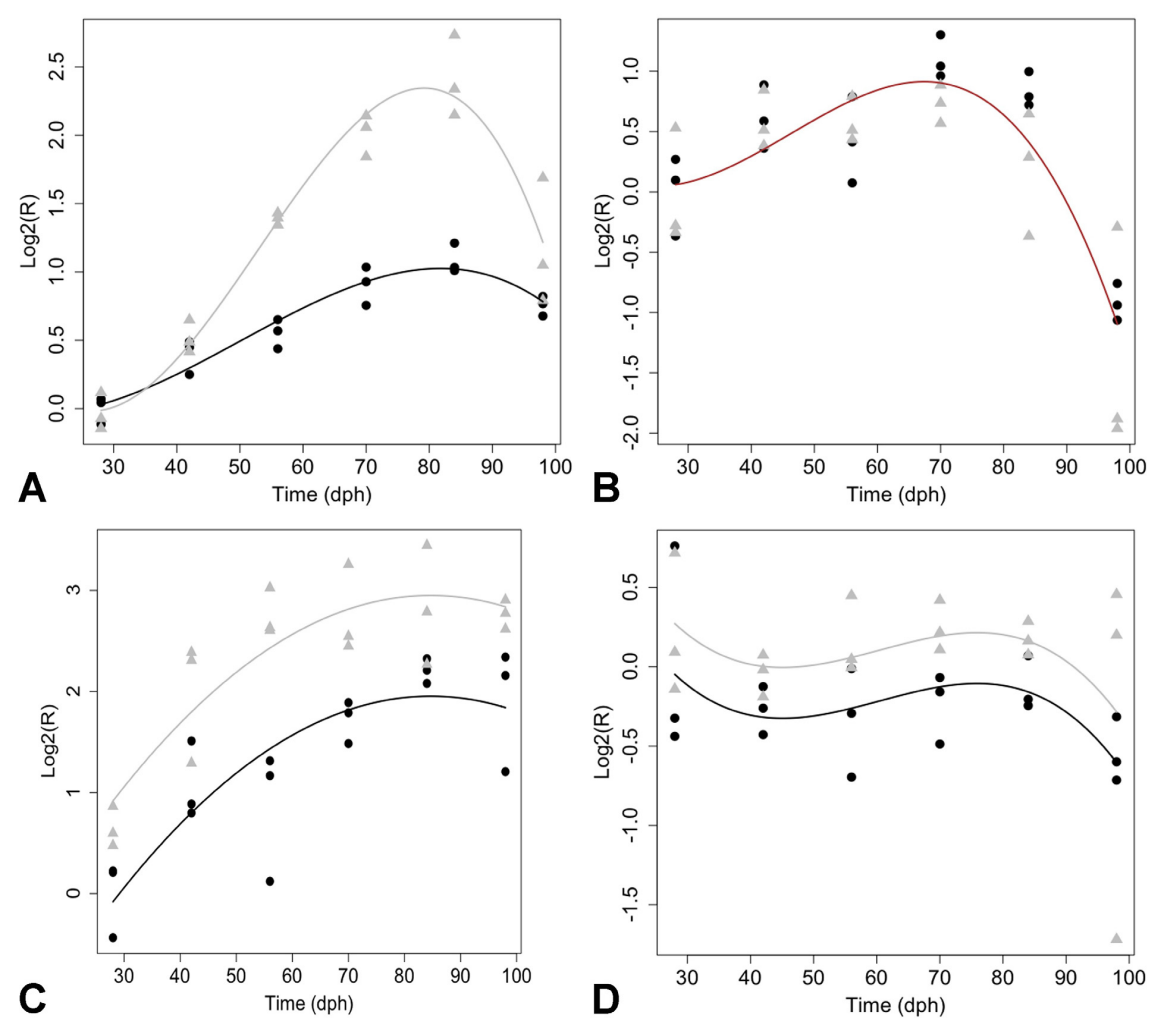
**Expression profiles generated by qPCR**. The figure shows profiles for hypothalamic-pituitary-interrenal axis genes: (A) *nr3c2*, (B) *nr3c1*, (C) *pomc*, and (D) *crhr1*. Axolotl larvae = black circles, tiger salamander larvae = gray triangles. Separate trend lines indicate statistically significant profiles for all genes except *nr3c1 *at the p = 0.05 level.

Some of the DEGs that were identified by microarray analysis suggest the HPI axis is differentially regulated between tiger salamander and axolotl larvae. For example, microarray analysis estimated higher transcript abundances for *nr3c2 *in tiger salamanders, while *nr3c1 *did not differ between the species. In adult mammals and presumably amphibians, glucocorticoids feedback to regulate brain development and function by binding to *nr3c1 *and *nr3c2*, which like thyroid hormone receptors, act as transcription factors. To verify transcript abundance estimates for these genes, and extend the analysis to two additional HPI axis genes (*crhr1, pomc*), quantitative real-time reverse transcription PCR (qPCR) was used to examine mRNA expression across a broader range of time points (28 - 98 dph; Figure 6). Both *nr3c1 *and *nr3c2 *showed a pattern of increasing transcript abundance with maximal levels attained at 70 dph for *nr3c1 *and 84 dph for *nr3c2*; after these times, levels decreased. Whereas *nr3c1 *showed the same pattern of expression in both species, *nr3c2 *abundance was two fold higher in tiger salamander larvae at 84 dph. A pattern of increasing transcript abundance was also observed across the larval period for *pomc *while *crhr1 *mRNA levels remained relatively flat. For both of these genes, mRNA levels were significantly higher in tiger salamander larvae. These qPCR results corroborate the earlier microarray results and show that HPI axis genes are more highly expressed in tiger salamanders during larval development.

**Figure 7:**
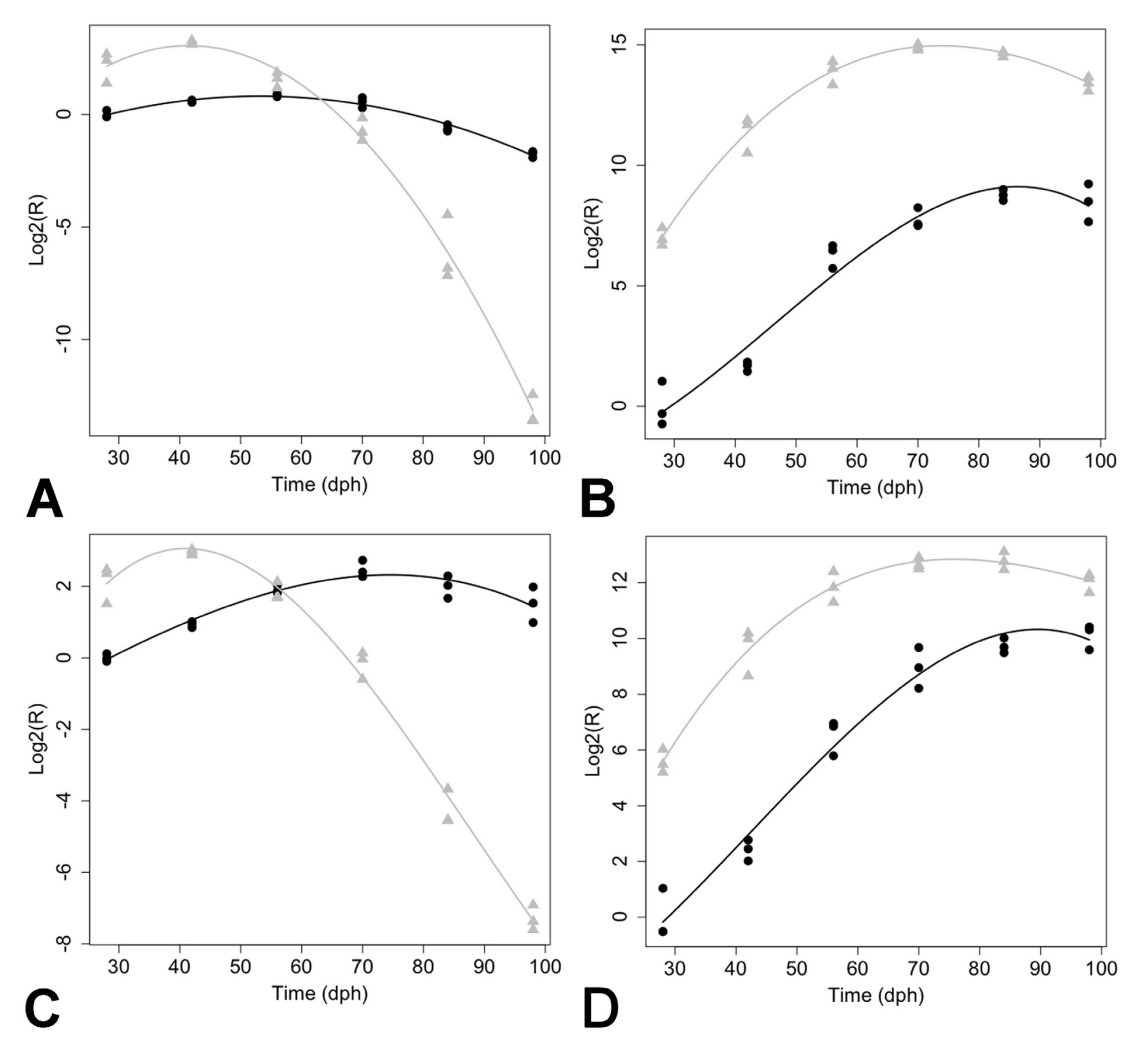
**Expression profiles generated by qPCR for hemoglobin genes**. (A) *hba*, (B) *hbd*, (C) *hbe1*, and (D), *hbg1*. Axolotl larvae = black circles, tiger salamander larvae = gray triangles. Separate trend lines indicate statistically significant profiles for all genes at the p = 0.05 level.

The microarray analysis also showed dramatic changes in hemoglobin mRNA abundances both within and between species. Presumably, these gene expression patterns reflect transcription within erythrocytes that were isolated from vasculature within the brain. The *Ambystoma *genome appears to encode multiple paralogs for almost all hemoglobin loci and these are expressed differently in some cases (unpublished data). For example, paralogs of *hba *in tiger salamander showed both increasing (SRV_00496_s_at) and decreasing (SRV_02508_x_at) patterns of mRNA abundance, while all axolotl *hba *paralogs showed flat or increasing patterns (Additional File 1). qPCR yielded a flatter profile for axolotl *hba *than was suggested by the microarray analysis, however qPCR replicated the dramatic decrease in mRNA abundance for tiger salamander *hba*, as well as the expression patterns of three additional hemoglobin loci for both species (Figure 7). Overall, the patterns suggest that axolotls and tiger salamanders similarly up regulate some of the same hemoglobin loci (*hbd, hba, hbg*) during the larval period, although levels were consistently higher in tiger salamander larvae. The only locus that was similarly down regulated was *hbz*. Interestingly, axolotls maintain higher embryonic-type hemoglobin (*hbe*) mRNA levels than tigers throughout the larval period, a pattern that is consistent with a paedomorphic mode of development.

## Discussion

This study used a functional genomics approach to detail larval brain transcription between the paedomorphic Mexican axolotl and metamorphic tiger salamander. The results show that larvae of these species have different transcriptional programs that are distinguishable in two important respects. First, although shared expression patterns were observed between the species, most of the genes that were identified as differentially expressed during the larval period showed species-specific patterns of expression. Gene expression was more similar between the species at earlier time points, with pronounced differences observed at 70 and 84 dph, which coincided with the onset of anatomical metamorphosis in a subset of the tiger salamander larvae. Second, the abundance of mRNAs tended to be higher for genes that were up regulated during tiger salamander development, relative to those that were up regulated during axolotl development. Approximately 31% of genes that could be reliably and directly compared between the species were differentially expressed and 84% of these showed higher mRNA abundances in tiger salamander larvae. Below, we discuss these primary results and explore their relationships to transcriptional programming that may correlate with metamorphic and paedomorphic modes of development.

Similar expression patterns were observed for 99% of the genes that were commonly differentially expressed in axolotl and tiger salamander larvae as a function of time. This suggests that some biological processes are regulated similarly between axolotl and tiger salamander larvae during development. For example, it is possible that some of the genes that function in the specification and proliferation of neuronal cell types are similarly expressed during development in both metamorphic and paedomorphic salamanders. Genes associated with vertebrate brain development such as *sox3 *[32], *msx1 *[33], and *npy *[34] significantly increased in abundance in both species. Also *clu*, a gene expressed at low levels in the central nervous systems of embryonic mice before increasing during postnatal life [35], was similarly up regulated during the axolotl and tiger salamander larval periods. Many aspects of brain development and function are highly conserved among vertebrates. If these functions depend upon conserved patterns of gene transcription, then similarities are expected whether a salamander follows a metamorphic or paedomorphic mode of development.

Although many DEGs were expressed similarly between the species, approximately four times as many were uniquely differentially expressed in tiger salamander larvae. It is possible that many of these gene expression differences represent transcriptional responses in tiger larvae that are necessary for metamorphosis. Three lines of evidence support this idea. (1) More genes were uniquely expressed in tiger larvae and the majority of these showed larger fold changes later in the larval period, when larvae were undergoing anatomical metamorphosis. (2) Hundreds of genes were differentially expressed between axolotls and tiger salamanders throughout larval development, including the earliest time point sampled (42 dph). (3) Genes and gene functions that are likely associated with later metamorphic regulation were identified as DEGs. For example, several genes that encode chromatin structure and modifying proteins were uniquely identified from tiger larvae or were differentially expressed between the species (*e.g. dnmt1*, *baz1a, smarca5*). Also, several genes that may function in post-transcriptional modification of chromatin proteins were identified as differentially expressed between the species (e.g. *sumo1, uba2, ube2e3, ube2I, ube2l3, ube2r2*). It is well established that metamorphosis in amphibians and insects requires programming events that activate new transcriptional programs [36]. Indeed, knocking out *smt3 *(homolog of *sumo1*) in *Drosophila*, a gene associated with chromatin remodeling by sumoylation, is known to extend the pupal stage and inhibit metamorphosis [37]. In addition to chromatin-associated genes, the expression of several genes involved in cellular metabolic processes (e.g. *cat, got2, adss, acp1, idh3g, ctps, eno3*) and hormone pathways (e.g. *sstr5, nr3c2, prl*) increased or were expressed at higher levels (*tef*) during tiger salamander development. While it is not unexpected to discover gene expression differences between closely related species, the results are intriguing because transcriptional output was generally higher in tiger salamanders, for the majority of loci that showed differential expression. Moreover, some of the genes that were differentially expressed were identified early in the larval period, well before the onset of morphological metamorphosis. This suggests that metamorphic and paedomorphic modes of development are distinct in a transcriptional sense at very early stages of ontogeny, perhaps tracing back to embryogenesis [16].

Paedomorphosis is a heterochonic term that is classically defined as a change in the timing of development that leads to the retention of ancestral, juvenile characteristics in adults of evolutionarily derived lineages [13]. The simplest model to explain such a pattern is a change that delays the overall rate of development. Patterns of gene expression were discovered that support the idea of developmental delay: axolotls maintained relatively constant *hbe *and *hba *transcript abundances, suggesting the maintenance of an embryonic hemoglobin expression profile throughout larval development. In comparison, transcripts for these genes declined precipitously midway during the larval period in tiger salamanders, perhaps coinciding with the initiation of early, metamorphic changes. However, developmental delay cannot explain all of the axolotl-tiger salamander expression differences. As was noted above, axolotls shared some expression patterns with tiger salamander larvae, which presumably present aspects of the ancestral metamorphic pattern. In addition, axolotls showed unique expression patterns not observed in tiger salamander larvae. Axolotls uniquely up regulated genes that are associated with vertebrate brain development and mammalian brain pathologies, including *ctss *and aging [38], *ogn *and pituitary cancer [39], and *cd69 *and Alzheimers [40]. Also, an expression difference was identified that supports the idea of a depressed HPT axis in axolotls [21,25]. *Tef*, a bZIP transcription factor implicated in the activation of mammalian TSHb [41], showed significantly lower abundances across all time points in axolotl larvae. Finally, consider the transcription of *nr3c1 and nr3c2 *during development. The glucocorticoid receptor (*nr3c1*) showed a dynamic temporal expression profile that was statistically indistinguishable in both species. Conversely, the mineralocorticoid receptor (*nr3c2*) was expressed at significantly lower levels in axolotl larvae. Thus, gene expression patterns in larval axolotls appear to be mosaic: some patterns are shared with tiger salamanders and some patterns are novel. Some of the novel patterns may be neutral in effect and fixed by genetic drift. However, in the case of transcription factors that may influence hypothalamic-pituitary development and activity (*tef, nr3c2*) or hemoglobins that allow for physiological adaptation to changing oxygen needs, the axolotl provides a model to study expression changes that have likely been selected to better suit large and reproductively competent "larval forms" for a totally aquatic life history.

In considering how discontinuous phenotypes evolve via Darwinian means, Gould [16] proposed an answer in the case of the axolotl:

"the problem of reconciling evident discontinuity in macroevolution with Darwinism is largely solved by the observation that small changes early in embryology accumulate through growth to yield profound differences among adults.... Delay the onset of metamorphosis and the axolotl of Lake Xochimilco reproduces as a tadpole with gills and never transforms into a salamander." 

Although once a controversial idea, it is accepted now that evolution can act on early stages of development to yield novel phenotypes [42]. There is a lengthy temporal disconnect between embryogenesis and the time a tiger salamander larva first shows morphological changes indicative of metamorphosis (after 56 DPH). During this time, there is ample time for genetic and environmental factors to affect brain development in ways that alter hypothalamic-pituitary activity late in the larval period. The results show that many genes in the brains of axolotl larvae are transcribed at lower levels than they are in tiger salamander larvae. These include genes that function in the regulation of hypothalamic-pituitary activities that orchestrate anatomical metamorphosis. This suggests the following hypothesis: an axolotl's failure to undergo metamorphosis late in the larval period traces to mechanisms that act early in development to broadly program transcription. This hypothesis can be tested by over-expressing tiger salamander genes in axolotl embryos that function to program gene expression in the brain during early development. Other hypotheses can be tested in axolotls to investigate mechanisms that direct brain development in a predictable manner, towards a hopeful monster outcome.

## Conclusion

This study shows that axolotl and tiger salamander larvae present different brain transcriptional programs and these programs diverge early in development. These early transcriptional differences include genes whose functions associate with a number of biological processes, including cell cycle, apoptosis, chromatin structure and remodeling, cellular metabolism, transcription, post-translational modification, neural development, and regulation of the HPI and HPT axes. Studies of other metamorphic and paedomorphic species of salamander are needed to disentangle species-specific gene expression responses from those that distinguish metamorphic and paedomorphic modes of development.

## Methods

### Study animals

A single fertilized clutch of *A. t. tigrinum *was obtained from Charles D. Sullivan Co. Inc and the *A. mexicanum *were sibs deriving from a Voss lab axolotl strain. Larvae from both species were reared individually following hatching in 40% Holtfretter's solution at 20-22°C and fed brine shrimp napulii (*Artemia *sp., Brine Shrimp Direct, Ogden, UT) twice daily for three weeks. After three weeks, larvae were fed California blackworms *ad libitum *(*Lumbriculus *sp., J.F. Enterprises, Oakdale, CA). At 28, 42, 56, 70, 84, and 98 dph, salamanders were anesthetized in 0.01% benzocaine and whole brains and attached pituitaries were flash-frozen in liquid nitrogen immediately following collection. Observations and measurements were collected on larval to monitor the progression of tiger larvae towards metamorphosis. Snout-vent-length (SVL) was recorded for the salamanders from which tissues were collected. A general linear model of the following form was fit to the SVL data: SVL_tij _= β_0 _+ S_t _+ T_i _+ (ST)_ti _+ T^2^_i _+ (ST^2^)_ti _+ ε_tij _where β_0 _corresponds to the intercept term for axolotls, S_t _corresponds to the intercept term for tiger salamanders, T_i _corresponds to the linear regression coefficient for axolotls, (ST)_ti _corresponds to the linear regression coefficient for tiger salamanders, T^2^_i _corresponds to the quadratic regression coefficient for axolotls, (ST^2^)_ti _corresponds to the quadratic regression coefficient for tiger salamanders, and ε_tij _corresponds to the error term of the jth individual from species t sampled at time i. Animal care and use was approved by the University of Kentucky Animal Care and Use Committee (IACUC protocols # 01087L2006 and #00907L2005).

### RNA isolation

Three tissue pools were developed for each time point. Whole brains and attached pituitaries were used because of the small brain size of early larvae, which yielded low amounts of RNA. Each tissue pool contained the brains of three different individuals. Total RNA was isolated using TRIzol (Invitrogen, Carlsbad, CA) and RNA samples were further purified using Qiagen RNeasy mini-columns. RNA samples were quantified via UV spectrophotometry (NanoDrop, ND-1000) and qualified via an Agilent BioAnalyzer (Agilent Technologies).

### Gene expression profiling

Genome-level expression profiling was conducted using a custom Affymetrix GeneChip [28-30]. Three replicate RNA pools for each of four time points (42, 56, 70, 84 dph) were labeled, hybridized, and scanned by the University of Kentucky Microarray Core Facility according to standard Affymetrix protocols. Additional gene expression profiling for selected genes was conducted for a broader range of time points (28, 42, 56, 70, 84, and 98 dph) using qPCR. Primers (Additional File 5) were designed using Primer3 [43]. When possible, axolotl and tiger salamander orthologs for each gene in Additional File 5 were aligned via BLAST to identify gene regions that corresponded to the same nucleotides covered by Affymetrix probe-sets. When the orthologs were not 100% identical in the target regions, separate primers were designed for each species (see Additional File 5). A BioRad iScript Select cDNA synthesis kit (Hercules, CA, USA) was used to synthesize cDNA from 1 μg of total RNA and primer efficiencies were estimated separately for axolotl and tiger salamander via linear regression on dilution series. A reference gene (*tif1; *probe-set L_s_at; Additional File 5) was demonstrated to be invariant across all species by time combinations and relative expression ratios were calculated according to Pfaffl [44]. All expression ratios are relative to the mean expression of axolotl at 28 dph and normalized to *tif1*. All PCRs were 10 μl reactions consisting of 4 ng cDNA, 16.4 ng of forward and reverse primers, and Roche FastStart Universal SYBR Master (Rox) Mix (Roche Diagnostics, Indianapolis, IN). PCRs were conducted on an Applied Biosystems StepOnePlus real-time PCR system. Reaction conditions were as follows: 10 minutes at 95°C, 40 cycles of 15 seconds at 95°C followed by 1 minute at 55°C, 15 seconds at 95°C, and 1 minute at 55°C. Melting curves were generated to ensure amplification of a single product for each reaction. All reactions were run on 48 well plates and blocked by sampling time and species (*i.e*., for a given time point, both species were present on the plate). At least two template free controls were present on each plate [45].

### Quality control and low-level analyses of the Ambystoma GeneChip

All arrays were subjected to quality control (QC) at the individual probe level by inspecting box-plots, histograms, pair-wise M versus A plots of replicate GeneChips, pseudo-images of probe level models, and an RNA degradation plot that allows for visualization of the 3' labeling bias across all GeneChips simultaneously [46,47]. Background correction, normalization, and expression summaries were obtained via the robust multi-array average (RMA) algorithm [48]. Two RMA expression matrices were generated separately for each species (see below) and a third RMA expression matrix was computed from all arrays from both species (see below). Upon implementing the RMA algorithm, the probe-set level data from each of these three matrices were subjected to further QC by inspecting pair-wise M vs. A plots of replicate GeneChips and examining correlation matrices among replicate GeneChips (minimum mean *r *for a given species by time point combination across all three of the RMA expression matrices = 0.989). Probe-sets from the two species-specific RMA matrices that were classified as "absent" on > 75% of the GeneChips were filtered [49].

### Identification of identical probe-sets

A total of 1604 (~33%) of the 4844 probe-sets on the *Ambystoma *GeneChip were designed from contigs that have predicted orthologs in axolotl and tiger salamander. The sequences of the probe-sets were used as queries in BLAST searches of axolotl and tiger salamander EST contigs [50]. BLAST alignments were used to extrapolate the number of mismatches (MM) between microarray probes designed to axolotl and orthologous EST contigs from tiger salamander, and vice versa. These data were then used to calculate the number of probes in each probe-set that had > 0 MM and the sum of MM across each probe-set. Probe-sets that had > 0 MM between species were filtered before conducting statistical analyses that directly compared expression values between axolotl and tiger salamander larvae (see below).

### Identification of differentially expressed genes

Two statistical approaches were used to identify DEGs. First, RMA matrices were generated for each species and quadratic regression [51] was used to identify genes that changed as a function of time. This approach also classified genes into nine different temporal profiles based on the values of the estimated regression coefficients (Figure 3; see also [52]). The "flat" profile describes genes that do not show transcript abundance changes (null results). The LU, LD, QLVU, and QLCD profiles described genes that show linear (LU, LD) or nonlinear (QLVU, QLCU, QLCD, QLVD) changes in transcript abundance across sample times. The QV and QC expression profiles described genes that show transient changes. Statistical correction for multiple testing was done separately for each species by evaluating α_0 _at a false discovery rate (FDR- [53]) of 0.05. α_1 _was set to 0.05. In addition to statistical criteria, genes were only retained if they exhibited ≥ 1.5 fold changes relative to 42 dph (baseline) at one or more of the other time points (56, 72, or 84 dph).

The second statistical approach used the global RMA matrix to directly compare axolotl and tiger salamander expression levels/profiles for genes known to exhibit zero sequence divergence in the regions encompassed by Affymetrix probe-sets (see above). This analysis was conducted using the maSigPro software package [54] that is available from bioconductor http://www.bioconductor.org for the R statistical computing environment http://www.r-project.org. In short, maSigPro was used to fit second order (*i.e*., quadratic) regression models, in which the species term is identified by a dummy variable, in a gene-by-gene manner. Correction for multiple testing was achieved by evaluating the over-all model P-values according to the algorithm of Benjamini and Hochberg [53] at an FDR of 0.05. A backward selection procedure was then used to eliminate non-significant (α = 0.05) terms from significant models. In order for genes from this analysis to be considered "identified" they had to meet the following criteria: (1) over-all model *P*-values lower than the FDR adjusted threshold, (2) significant species, time × species, or time^2 ^× species terms, (3) an *R*^*2 *^≥ 0.50, and (4) a ≥ 1.5 fold difference between axolotl and tiger salamander at one or more of the sampling times (42, 56, 70, or 84 dph).

### Identification of statistically enriched biological processes

To identify biological processes that were statistically enriched in our lists of DEGs, we conducted EASE analyses using the database for annotation visualization and integrated discovery (DAVID)[55]. For all analyses, the 3728 genes on the *Ambystoma *GeneChip with established orthologies to humans were used to generate expected values (*i.e*., as the background). The count threshold was always set to two and the EASE threshold was always set to 0.05. The list of significant GO terms was manually inspected to remove redundant terms.

### Statistical analysis of the qPCR data

General linear models were fit to qPCR estimates of mRNA abundance to determine if gene expression differed in magnitude and/or temporal profile between the species. These models took the form: Log_2_(R)_tij _= β_0 _+ S_t _+ T_i _+ (ST)_ti _+ T^2^_i _+ (ST^2^)_ti _+ T^3^_i _+ (ST^3^)_ti _+ ε_tij _where β_0 _= the intercept term for axolotl, S_t _= the intercept term for tiger salamander, T_i _= the linear regression coefficient for axolotl, (ST)_ti _= an additional linear regression coefficient for tiger salamander, T^2^_i _= the quadratic regression coefficient for axolotl, (ST^2^)_ti _= an additional quadratic regression coefficient for tiger salamander, T^3^_i _= the trinomial regression coefficient for axolotl, (ST^3^)_ti _= an additional trinomial regression coefficient for tiger salamander, and ε_tij_= the error term associated with jth RNA pool from species t and time i. When necessary, these models were simplified via a backward selection scheme that removed non-significant terms (*P *> 0.05).

## Authors' contributions

RBP designed the microarray experiment, conducted statistical and bioinformatics analyses, analyzed data and interpreted results, and contributed to the writing of the manuscript. MAB collected morphometric measurements and tissue samples from *A. mexicanum*, isolated RNA and performed qPCR, analyzed and interpreted data, and contributed to the writing of the manuscript. JJS conceived the temporal sampling design and collected morphometric measurements and tissue samples from *A. t. tigrinum*. SP helped with bioinformatic analyses. SRV analyzed data and interpreted results, and led the manuscript writing effort. All authors have read and approved the final manuscript.

## Supplementary Material

Additional file 1 **Differentially expressed genes identified from axolotl and tiger salamander larvae**. Table contains 134 total probesets that correspond to 108 different genes identified from larval axolotls and tiger salamanders. BLASTx searches were performed to identify presumptive human orthologs. The regression profile for each gene is presented. Click here for file

Additional file 2 **Genes uniquely identified from axolotl larvae**. Table contains 85 total probesets that correspond to 76 different genes identified from larval axolotls. BLASTx searches were performed to identify presumptive human orthologs. The regression profile for each gene is presented. Click here for file

Additional file 3 **Genes uniquely identified from tiger salamander larvae**. Table contains 332 probesets that correspond to 292 different genes identified from a direct comparison of gene expression between the brains of larval axolotls and tiger salamanders. BLASTx searches were performed to identify presumptive human orthologs. The regression profile for each gene is presented. Click here for file

Additional file 4 **Differentially expressed genes identified from axolotl and tiger salamander larvae**. Table contains 419 probesets that correspond to 409 different genes identified from a direct comparison of gene expression between the brains of larval axolotls and tiger salamanders. BLASTx searches were performed to identify presumptive human orthologs. Click here for file

Additional file 5 **Primer sequences used for qPCR assays**. Table of genes and corresponding primer sequences that were used to accomplish qPCR. For the "Species" column, "Both" denotes genes with identical sequences between A. *mexicanum *and *A. t. tigrinum*, "Tiger Only" denotes genes for which sequence information was only available from *A. t. tigrinum *"Axolotl Only" denotes genes for which sequence information was only available from *A. mexicanum*, "Tiger" denotes primers that were designed from A. t. tigrinum sequence, and "Axolotl" denotes primers that were designed from *A. mexicanum *sequence. +/- denotes the direction of a given primer. Click here for file
